# Studying the microbial, chemical, and sensory characteristics of shrimp coated with alginate sodium nanoparticles containing *Zataria multiflora* and *Cuminum cyminum* essential oils

**DOI:** 10.1002/fsn3.3261

**Published:** 2023-02-10

**Authors:** Mahmoud Osanloo, Zahra Eskandari, Elham Zarenezhad, Hajar Qasemi, Amene Nematollahi

**Affiliations:** ^1^ Department of Medical Nanotechnology, School of Advanced Technologies in Medicine Fasa University of Medical Sciences Fasa Iran; ^2^ Student Research Committee Fasa University of Medical Sciences Fasa Iran; ^3^ Noncommunicable Diseases Research Center Fasa University of Medical Sciences Fasa Iran; ^4^ Department of Food Safety and Hygiene, School of Health Fasa University of Medical Sciences Fasa Iran

**Keywords:** active packaging, alginate, cumin, edible coating, seafood, thyme

## Abstract

Retardation of quality loss of seafood has been a new concept in recent years. This study's main objective was to evaluate the microbial, chemical, and sensory attributes of shrimp coated with alginate sodium nanoparticles containing *Zataria multiflora* and *Cuminum cyminum* essential oils (EOs) during refrigerated storage. At the end of storage time (15 days storage at 4°C), the pH, thiobarbituric acid reactive substances (TBARS), and total volatile basic nitrogen (TVBN) amounts in shrimps coated with the alginate nanoparticles were 7.62, 1.14 mg MDA/kg, and 117 mg/100 g which were significantly (*p* < .05) lower than the control groups. The count of all bacteria groups was also lower in this treatment, which was 2–2.74 Log CFU/mL on day 15 of cold storage. This combined treatment also obtained the highest sensory scores (around 7) and the lowest melanosis score (2.67) due to the effective delaying microbial and oxidation activities. Therefore, this edible coating could substantially retard microbial and chemical changes and improve the organoleptic properties of shrimp under refrigerated storage.

## Graphical abstract

Edible coatings have many advantages compared with synthetic packaging. Adding natural substances such as EOs to edible coatings increase the preservation of shrimp. Adding EO in the form of nanoparticles reduces the adverse effects of sensory properties.

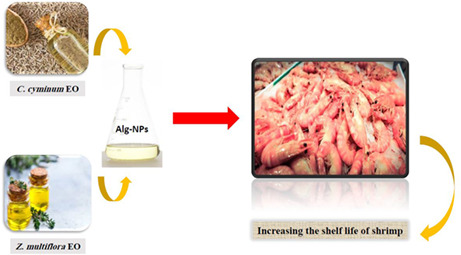

## 1 INTRODUCTION

Shrimp (*Litopenaeus vannamei*), one of the most important seafood commodities, is a valued food product due to its nutritional benefits and delicacy (Dehghani, Hosseini, Golmakani, et al., 2018; Dehghani, Hosseini, & Regenstein, 2018). The health benefits of shrimp mainly result from the existence of high concentrations of polyunsaturated fatty acids (PUFA), carotenoids (such as beta‐carotene and astaxanthin), fat‐soluble vitamins, phospholipids, and cholesterol (Gulzar et al., 2020; Wan Norhana et al., 2010). However, despite the high nutritional value of this food product, it spoils easily (even in cold storage). Subsequently, the shelf life of shrimp is restricted because of oxidation and microbial mechanisms (Lin et al., 2022) as well as the occurrence of melanosis (black spots) (Lama‐Muñoz et al., 2021). Moreover, these reactions have negative effects on organoleptic properties (such as texture, color, flavor, and overall acceptance) of shrimp (Na et al., 2018).

To this end, increasing the shelf life of food, including shrimp, has attracted much attention and significantly influenced the food industry's economic and health aspects (Na et al., 2018). Frozen storage and using some artificial or chemical preservatives are the best preservation method to increase the shelf life of shrimp. However, repeated freezing and thawing reduce the quality of shrimp. Moreover, given the toxic fate of some chemical additives and consumers' preference to buy foods containing natural additives, there is a need to use safer preservation methods (Alparslan et al., 2016; Arancibia et al., 2015; Zhang et al., 2015).

Essential oils (EOs) are natural ingredients added to food products as flavoring, antioxidants, and antimicrobial agents (Noorpisheh Ghadimi et al., 2020). The antimicrobial and antioxidant properties of EOs concerning food spoilage and pathogenic microorganisms have been proven by several studies (Abbasi et al., 2021; Hajlaoui et al., 2010; Mojaddar Langroodi et al., 2021; Moosavi‐Nasab et al., 2016).

*Zataria multiflora* Boiss (Lamiaceae family), known as thyme‐e‐Shirazy, is an aromatic herb that grows in several parts of Asia, such as southern Iran. This shrub has been applied as an antiseptic, antispasmodic, and anti‐inflammatory agent in traditional medication. *Zataria multiflora* is also commonly consumed as a flavoring and antimicrobial agent in home applications and the food industry (Hashemi et al., 2022). *Cuminum cyminum* L. (Apiaceae family), known as cumin, is also a small annual herbaceous plant native to Egypt and southeastern Iran. It has an intense and distinct taste due to having compounds like cuminaldehyde (Homayonpour et al., 2021). However, the straight addition of EOs into the food is mostly restricted due to several limiting factors, including the creation of strong taste, low solubility in water, high volatility, and probable reactions with other food ingredients (Zhang et al., 2021).

Incorporating EOs into edible films and coating (lipids, proteins, or polysaccharides) is a promising approach to solving these problems. Therefore, they remain around the product for a long time to have the best antimicrobial and antioxidant effectiveness during storage (Zarei et al., 2015). Alginate is a linear polyanionic biopolymer composed of β‐d‐mannuronic acid and α‐l‐guluronic acid parts (Yousefi, Azizi, et al., 2018; Yousefi, Farshidi, & Ehsani, 2018). It is naturally derived from brown seaweed (Phaeophyceae family). Besides the impermeability of alginate with oils and its poor water vapor barrier properties, alginate shows potentials like forming gels, spheres, and micro‐ and nanoparticles (Gheorghita Puscaselu et al., 2020).

Natural food packaging materials containing nanoparticles possessing active and intelligent traits have the ability to improve some of the food sector concerns. These materials could extend the shelf life of foods, develop food safety, and decline the amount of food waste resulted from deterioration. There is no regulatory construction for nanoparticle usage in packaging materials. However, using the metallic nanoparticles in active and intelligent packaging in the European Union (EU) is disallowed (except titanium nitride in plastic bottles). Nevertheless, nanoparticles could also be fabricated from nonmetals like organic materials such as proteins and polysaccharides as well as plant extracts (like EOs). They are food grade nanoparticles existing naturally or have been made in the nano size from food‐compatible materials. There is a higher public acceptance concerning these nanoparticles in comparison with metal nanoparticles because of their natural fate and no toxicity. The application of these nanoparticles is a solution to the intrinsic toxicity of metallic ones. There are numerous different food and plant extracts that possess antimicrobial characteristics and have the potential to be incorporated into food packaging (Hannon et al., 2015). Nowadays, biopolymer nanoparticles have attracted attention due to their inherent biological properties and size‐dependent features such as higher area and improvement effectiveness (Kraśniewska et al., 2020).

Many studies have been conducted on the effects of edible coatings and films combined with different EOs on several food products to increase their shelf life (Abdollahzadeh et al., 2021; Mojaddar Langroodi et al., 2021; Yousefi et al., 2022). However, to the best of our knowledge, there is no study investigating the combined incorporation of *Z. multiflora* and *C. cyminum* to alginate nanoparticles for the active packaging of shrimp. The main aim of the present study was thus to investigate the microbial, chemical, and sensory characteristics of shrimp coated with sodium alginate nanoparticles containing *Z. multiflora* and *C. cyminum* EOs during 15 days of storage at 4°C.

## 2 MATERIALS AND METHODS

### 2.1 Materials

The freshly caught shrimp were obtained from a local market in Fasa (Fars province, Iran) and brought to the laboratory with ice. After properly washing shrimps, they were kept at −18°C until testing. All culture media, including plate count agar (PCA), cetrimide fucidin cephaloridine agar (CFC), de Man‐Rogosa‐Sharpe agar (MRS), and violet red bile glucose (VRBG), as well as peptone water (PW), Tween 20 and Tween 80 were provided by Merck Company (Merck, Darmstadt, Germany). Sodium alginate and CaCl_2_ were also obtained from Sigma Company (Sigma‐Aldrich, Germany). *Zataria multiflora* and *C. cyminum* EOs were purchased from Zardband Pharmaceuticals and Tabibdaru companies (Iran), respectively. Distilled water was used in all tests.

### 2.2 GC–MS analysis

The gas chromatography device used was Agilent 6890 with column length, inner diameter, and layer thickness of 0.25 mm, 0.25 mm, and 0.25 μm, respectively (type BPX5). The process was described in our previous study. Briefly, to recognize the components of the EOs of each plant (*Z. multiflora* and *C. cyminum*), 1 μL of the sample diluted by *n*‐hexane was injected into the GC/MS system. The temperature program of the column was set as follows: the primary temperature of the oven was 50°C and stopped at this temperature for 5 min. Then the temperature increased to 300°C, and 3 min was held at this temperature. Thus, the response time was 75 min. The temperature of the injection part was 250°C as a 1:35 split. Helium was used as carrier gas with a course rate of 0.5 mL/min. The mass spectrometer applied was the Agilent 5973 model with ionization voltage, ionization method, and ionization source temperature of 70 electron volts, EI, and 220°C, respectively. The scan range of mass spectra was adjusted from 40 to 500. The spectra were identified using their inhibition index, the standard compounds' mass spectra, and the evidence in the apparatus library (Mojaddar Langroodi et al., 2021).

### 2.3 Preparation of nanoparticles

*Zataria multiflora* EO (0.125%), *C. cyminum* EO (0.125%), tween 20 (0.2%), and tween 80 (0.1%) were mixed (2000 rpm, room temperature, 3 min). First, 50 mL of alginate solution was added (0.25% w/v) and stirred for 5 min. Then, 50 mL of CaCl_2_ solution (0.06% w/v) was added. Finally, the obtained solution was stirred for 40 min to form nanoparticles (Alg‐NPs treatment). Also, nanoparticles without EO addition (Alg‐free treatment) were prepared similarly.

### 2.4 Characterization of nanoparticles

A DLS device (SZ‐100 series, HORIBA Scientific, Japan) was used to check the size, polydispersity index (PDI), and zeta potential of the prepared nanoparticles. ATR‐FTIR was also used to confirm the loading of EOs inside the nanoparticles. The spectra of *Z. multiflora* EO, *C. cyminum* EO, Alg‐free, and Alg‐NPs (from 400 to 4000 cm^−1^) were recorded. DPPH test was used to check the antioxidant properties of nanoparticles. A solution of 0.3 mM DPPH in ethanol was first prepared. Then, 150 μL of DPPH solution and 50 μL of serial dilutions were poured into each well of the plate (Osanloo et al., 2021). The plate was then incubated for 30 min in a dark environment, and the optical density (OD) was read at 517 nm. The antioxidant property was calculated according to Equation (1):

(1) $$\left(\mathrm{OD}\;\text{Control}-\mathrm{OD}\;\text{sample}\right)/\left(\mathrm{OD}\;\text{Control}\times 100\right).$$

### 2.5 Shrimp coating

Shrimp samples were classified into three different categories, including (a) uncoated sample (control), (b) sample treatment with nanoparticles alginate coating (Alg‐free), and (c) sample treatment with alginate nanoparticles containing *Z. multiflora* and *C. cyminum* EOs (Alg‐NPs). The treatment samples of shrimp (Alg‐free and Alg‐NPs) were soaked in the related liquid coating solutions for 5 min. After drying of coated shrimp in the environment, they were carefully placed in a polyethylene box and stored at 4°C for 15 days. Microbiological, chemical, and sensory analyses were performed at 3‐day intervals to determine the quality of coated shrimp samples.

### 2.6 Chemical analysis

#### 2.6.1 pH

According to Liu et al. (2020), 10 g of minced shrimp was mixed with 90 mL of distilled water for 30 s, and then the pH of the obtained solution was measured using a pH meter (3510 pH meter, GENWAY) at room temperature (Liu et al., 2020).

#### 2.6.2 Thiobarbituric acid reactive substances (TBARS)

The TBARS test was conducted regarding Pikul et al. (1989) method with slight modification (Pikul et al., 1989). This test is usually performed to determine secondary oxidation products, that is, Malondialdehyde (MDA) (Mojaddar Langroodi et al., 2021). First, 200 mg of minced shrimp sample was blended with a little amount of 1‐butanol, and the volume of the obtained mixture was increased to 25 mL by the same solvent. Then, 10 mL of trichloroacetic acid (0.2%) reagent was added to 5 mL of the previously obtained mixture and kept in a hot water bath (95°C, 2 h). After cooling the resulting solution at ambient temperature, its absorbance was measured at 532 nm. TBARS value was calculated, as mg MDA/kg shrimp, using Equation (2):

(2) $$\text{TBARS}=\frac{50\times \left(A-B\right)}{m},$$

where *A*, *B*, and *m* are the absorbance of the sample solution, absorbance of the control solution, and weight of minced shrimp (mg).

#### 2.6.3 Total volatile basic nitrogen (TVBN)

To measure the TVBN amount, 10 g of minced shrimp was mixed with 2 g of magnesium oxide (MgO) and 250 mL of distilled water and a droplet of silicone (as an antifoam agent). Then, the obtained solution was poured into a tube containing 20 mL of boric acid (3% aqueous solution) in the presence of methyl red and methylene blue (as the indicator). After that, the solution was titrated with hydrochloric acid (HCl 0.01 N). Finally, the TVBN amount was calculated by the volume of consumed HCl and was stated as mg/100 g shrimp (Goulas & Kontominas, 2005).

### 2.7 Microbiological analysis

The count of lactic acid bacteria (LAB), coliform, *Pseudomonas*, and aerobic mesophilic was investigated during refrigerated storage. For this purpose, 1 g of minced shrimp was aseptically homogenized with 9 mL of peptone water (0.1%) using a stomacher (Labtron, UK) for 2 min. In the case of all microbial assays, decimal serial dilutions were made in peptone water (1.0%) of the homogenized prepared solutions. The count of the total aerobic mesophylls was performed by inoculating the sample on PCA culture and incubating it for 24 h at 32°C. The CFC agar was used to calculate Pseudomonas numbers (incubated for 24 h at 20°C). LAB was enumerated on MRS agar after incubation for 1 day at 37°C. Coliforms were counted on VRBG agar after incubation for 24 h at 37°C (Abbasi et al., 2021; Mojaddar Langroodi et al., 2021).

### 2.8 Sensory evaluation

The sensory properties of shrimp samples were examined by seven trained panelists who do not have an allergy to shrimp and other seafood. The assessors did not know about the experimental investigation, and the shrimp samples were provided with a numerical code (with no special meaning). The assessment of texture, odor, color, and overall acceptability was performed using a 9 points hedonic test (9 = very favorable and 1 = very unfavorable). In addition, the assessors' satisfaction with being involved has been considered (Liu et al., 2020).

### 2.9 Melanosis assessment

Shrimp samples' melanosis (black spots) was visually investigated by the same 7 trained panelists based on a scoring system during refrigerated storage (Alparslan et al., 2016).

### 2.10 Statistical analysis

In the present study, all tests were carried out in triplicate, and all data are expressed as the average (mean) ± standard deviation (SD). All obtained data were evaluated using a two‐sample mean comparison using STATA software (version 11). *p* < .05 was reflected as a significance cutoff.

## 3 RESULT AND DISCUSSION

### 3.1 Chemical composition of *Zataria multiflora* and *Cuminum cyminum* EOs

Active chemical components in *Z. multiflora* and *C. cyminum* EOs quantified by GC–MS are shown in Table 1. In total, 15 and 8 compounds were identified in *Z. multiflora* EO and *C. cyminum* EO, respectively. As depicted in this table, thymol (25.2%) and carvacrol (30.2%) are the main components of *Z. multiflora* EO, while cuminic aldehyde (30.8%) is the main compound of *C. cyminum* EO. Similar to our results, Shafiee and Javidnia (1997) reported that thymol and carvacrol are the main components of *Z. multiflora* EOs (Shafiee & Javidnia, 1997). Also, Hajlaoui et al. (2010) reported that cuminic aldehyde (39.48%) is the main compound of *C. cyminum* EOs (Hajlaoui et al., 2010). However, the concentration of carvacrol in Azizkhani et al. (2013) and Shafiee and Javidnia (1997), as the main compound of the *Z. multiflora* EO were much higher than our study, which was 71.12% and 61.29%, respectively (Azizkhani et al., 2013; Shafiee & Javidnia, 1997). These differences in EO components could be related to geographical conditions, climatic changes, age and region of the plant, harvesting season, and EO extraction method (Azizkhani et al., 2013).

**TABLE 1:** Identified compounds with >1% in *Cuminum cyminum* and *Zataria multiflora* EOs using GC–MS analysis.

RT^a^	Compound	*Cuminum cyminum*			*Zataria multiflora*		
		Area	%	RI^b^	Area	%	RI
9.4	α‐Pinene	37,379,726	1.2	932	230,827,972	3.6	932
11.3	β‐Pinene	367,096,282	11.6	979	‐	‐	‐
12.0	β‐Myrcene	‐	‐	‐	75,429,639	1.2	988
13.1	α‐Terpinene	‐	‐	‐	92,129,256	1.4	1014
13.6	*p*‐Cymene	573,807,048	18.1	1024	‐	‐	‐
13.6	o‐Cymene	‐	‐	‐	684,964,276	10.7	1022
13.7	β‐Phellandrene	31,642,040	1.0	1029	‐	‐	‐
13.8	1,8‐Cineole	‐	‐	‐	159,004,914	2.5	1026
15.2	ɣ‐Terpinene	486,212,840	15.4	1059	391,836,818	6.1	1054
17.1	Linalool	‐	‐	‐	126,174,339	2.0	1095
23.7	Carvacrol methyl ether	‐	‐	‐	84,815,310	1.3	1241
23.8	Cuminic aldehyde	974,724,263	30.8	1239	‐	‐	‐
25.6	1‐Isopropylidene‐3‐n‐butyl‐2‐cyclobutene	209,723,917	6.6	1271	‐	‐	‐
26.5	Thymol	‐	‐	‐	1,608,717,376	25.2	1289
27.1	Carvacrol	‐	‐	‐	1,930,172,282	30.2	1298
28.7	Thymyl acetate	‐	‐	‐	75,349,373	1.2	1349
29.5	Carvacryl acetate	‐	‐	‐	129,791,198	2.0	1372
31.3	Caryophyllene oxide	‐	‐	‐	87,065,417	1.4	1582
32.1	*trans*‐Caryophyllene	‐	‐	‐	140,081,621	2.2	1583
33.5	α‐Acoradiene	51,304,216	1.6	1464	‐	‐	‐
37.8	Aromadendrene	‐	‐	‐	76,499,808	1.2	1641

^a^ Retention time (min).

^b^ Retention index.

### 3.2 Characterization of nanoparticles

**FIGURE 1:**
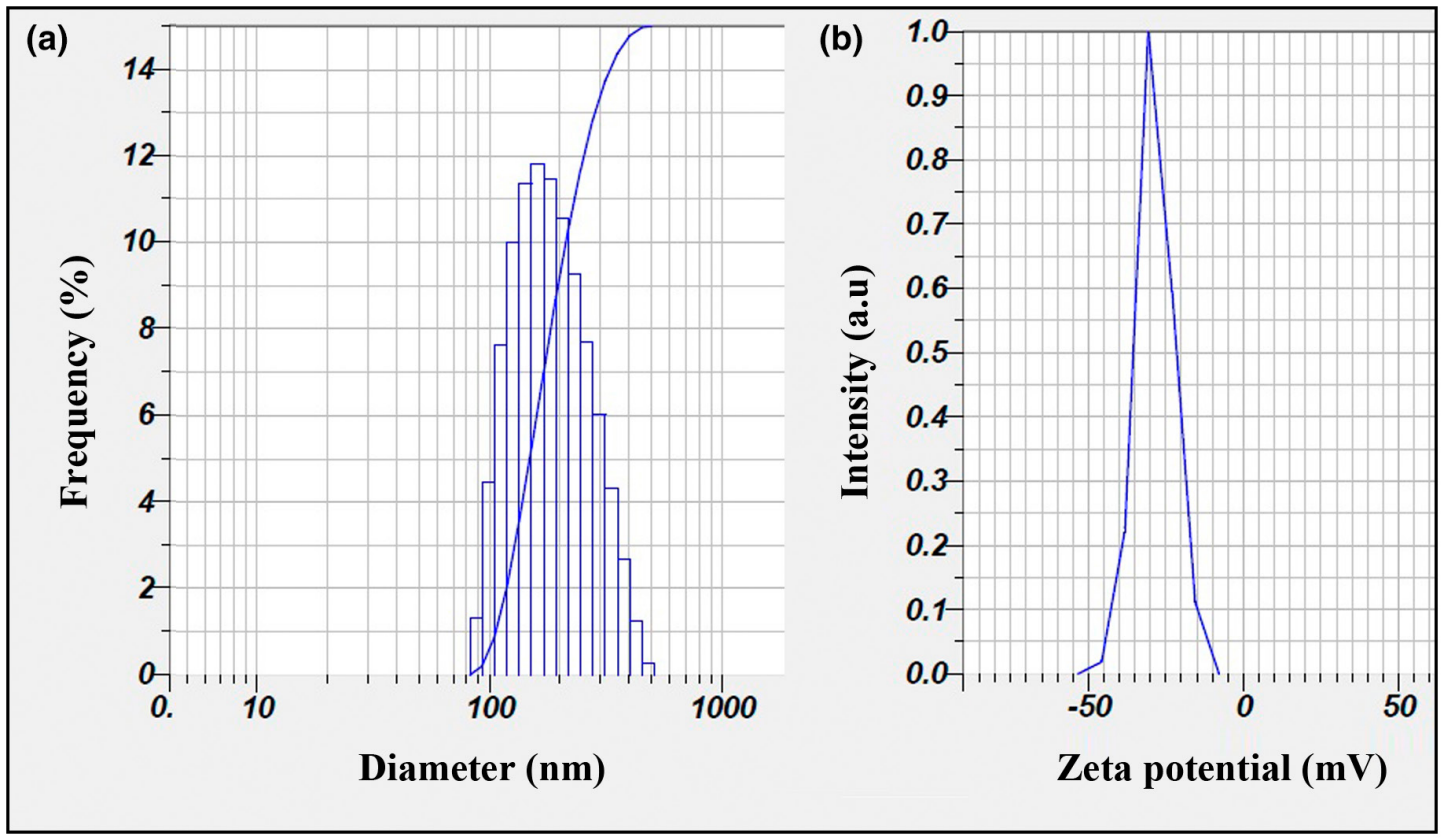
DLS analysis, 195 ± 7 nm (a) and Zeta potential, −29 ± 2 mV (b) of the alginate nanoparticles containing *Zataria multiflora* and *Cuminum cyminum* EOs.

DLS analysis and potential zeta profile of nanoparticles are depicted in Figure 1. The size, PDI, and zeta potential of nanoparticles were 195 ± 7 nm, 0.109, and −29 ± 2 mV, respectively. The wavelength of the light is much longer than the size of the nanoscale particles, so it does not contain any light angle and makes the nanoparticles appear clear or slightly hazy (Abbasi et al., 2021). In the study by Masoumi et al. (2016), the average particle size of the *Z. multiflora* EOs' nanoemulsion was 66.5 nm, which was lower than the results of this study (Masoumi et al., 2016). Conversely, Gahruie et al. (2017) reported that the particle size of nanoemulsion (oil in water) of *Z. multiflora* EOs (210.5 nm) was higher than the results of the present study. This difference could be attributed to the differences in the ultrasound intensity and preparation time (Gahruie et al., 2017).

Alginate, a natural anionic polymer derived from brown seaweed, has been widely used because of its biocompatibility, low toxicity, reasonably low price, and slight gelation using divalent cations like Ca^2+^ (Lee & Mooney, 2012). The first alginate particles for encapsulation were developed in 1980 (Lim & Sun, 1980). Advances in nanotechnology provide many synthetic protocols to produce polymer nanoparticles with dimensions below 100 nm. Polymeric nanoparticles have attracted great consideration because of the diversity of their characteristics in many applications (Ghanbariasad et al., 2021). The great surface‐to‐volume proportion of nanoparticles is related to significant physicochemical characteristics compared with their macroscale counterparts (Geetha et al., 2016). Sodium alginate has wide applications in the food industry, including beverages, dairy products, frozen products, salads, canned products, sauces, dessert gels, and puddings. It is also applied as an edible film and coating in many food products (Gheorghita Puscaselu et al., 2020). The possible reason for using alginate nanoparticles could be because alginate nanoparticles can contain many types of natural antimicrobial and antioxidant compounds, including EOs, enzymes, drugs, and other agents (Paques et al., 2014). To the best of our knowledge, there is no research regarding the alginate nanoparticles containing both *Z. multiflora* and *C. cyminum* EOs.

### 3.3 Attenuated Total Reflectance‐Fourier Transform InfraRed Spectroscopy (ATR‐FTIR)

**FIGURE 2:**
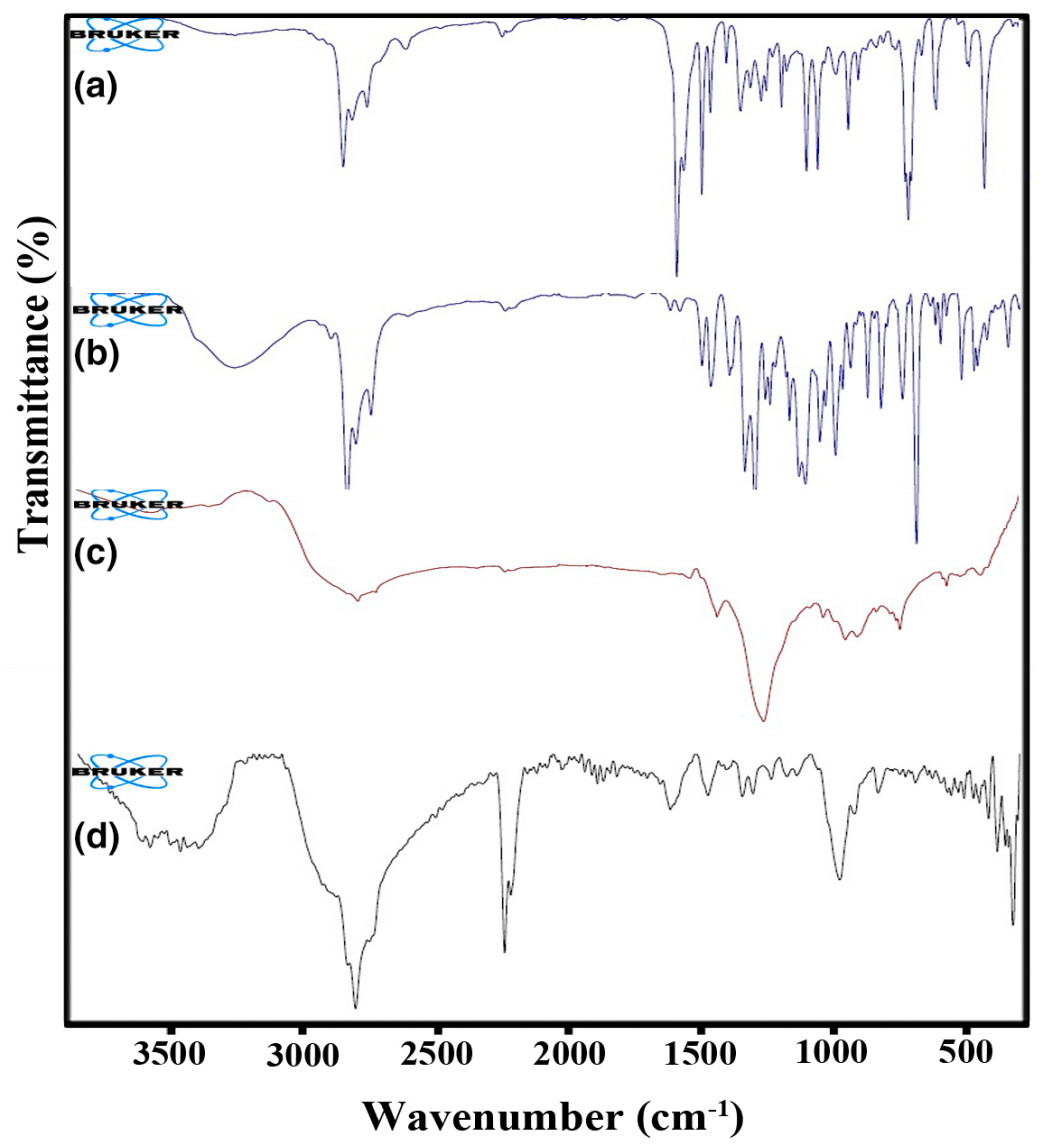
ATR‐FTIR spectra of EOs of *Cuminum cyminum* (a), *Zataria multiflora* (b), free alginate nanoparticles (c), and alginate nanoparticles containing the EOs (d).

ATR‐FTIR analysis was used to confirm the loading of EO inside the nanoparticles. Figure 2 shows ATR‐FTIR spectra of *C. cyminum* and *Z. multiflora* EOs (a and b), free alginate nanoparticles (c), and alginate nanoparticles containing *Z. multiflora* and *C. cyminum* EOs (d). The ATR‐FTIR spectrum of *C. cyminum* EO showed that the broad peak at 3368 cm^−1^ is related to the stretching vibration of OH. The bands at 2960, 2927, and 2870 cm^−1^ showed –the CH stretching vibration of Sp^3^ in alkanes. Besides, the bands at 2870 and 2723 cm^−1^ indicate C‐H of aldehyde. The characteristic bands at 1701 and 1675 cm^−1^ are assigned to the stretching vibration of carbonyl in aldehyde and ketones in EO. These strong peaks represented a high amount of aldehydes in the *C. cyminum* EO. The bands at 1575 and 1460 cm^−1^ showed the C=C vibration of the aromatic compounds. The peak at 1074 cm^−1^ is attributed to C‐O stretching vibration. The band at 948 cm^−1^ is related to C‐H bending absorption, and the strong peak at 827 cm^−1^ is assigned to benzene rings' C‐H vibration absorption. The peak at 687 cm^−1^ corresponds to the vibration absorption of alkenes.

Regarding the ATR‐FTIR spectrum of *Z. multiflora* EO, the broadband at 3200–3500 cm^−1^ can be attributed to the stretching vibration of the hydroxyl group due to hydrogen bonding in phenolic and alcoholic compounds in EO, the band at 3019 cm^−1^ can be related to stretching vibration of CH in sp^2^ groups and the bands at 2959, 2925, and 2969 cm^−1^ can be corresponded to stretching vibration of CH in sp^3^ groups, The band at 1738 and 1708 cm^−1^ can be attributed to stretching vibration carbonyl groups. The bands at 1088 and 1058 cm^−1^ displayed stretching vibration of the C‐O groups. The ATR‐FTIR spectrum of the free alginate nanoparticles showed that the broadband between 3200 and 3691 cm^−1^ could be related to the stretching vibration of hydroxyl groups in tweens, water, and alginate. The band at 1660 cm^−1^ can be related to the stretching vibration of the carbonyl group in tween. Two bands at 1558 and 1379 cm^−1^ related to carbonyl groups' symmetric and asymmetric stretching vibration. The band at 1115 and 1089 cm^−1^ can be attributed to the stretching vibration of C‐O groups.

In the case of alginate nanoparticles containing *Z. multiflora* EO and *C. cyminum* EOs, the ATR‐FTIR spectrum of nanoparticles containing *Z. multiflora* EO and *C. cyminum EO* showed a broad peak at about 3299–3731 cm^−1^, related to OH groups, due to hydrogen bonding in EO, alginate, and water. The band at 2925 cm^−1^ is attributed to C–H stretching vibration. The bands at 2345 and 2352 can be related to CO_2_, and the bands at 1771 and 1733 cm^−1^ are allocated to the stretching vibration of the carbonyl group in aldehyde in *C. cyminum* EO, tween, and alginate. The peaks at 1591 and 1352 cm^−1^ correspond to the symmetric and asymmetric stretching vibration of C=O groups. The strong peak at 1091 cm^−1^ is attributed to the reaction between the carboxyl and Calcium ion (CO‐Ca‐CO group structure), which enhanced C‐O vibration. This characteristic band demonstrated ionic crosslinking. The presence of other bands in EOs and alginate nanoparticles confirmed the successful loading of EOs in the prepared alginate nanoparticles.

### 3.4 DPPH free‐radical scavenging activity

**FIGURE 3:**
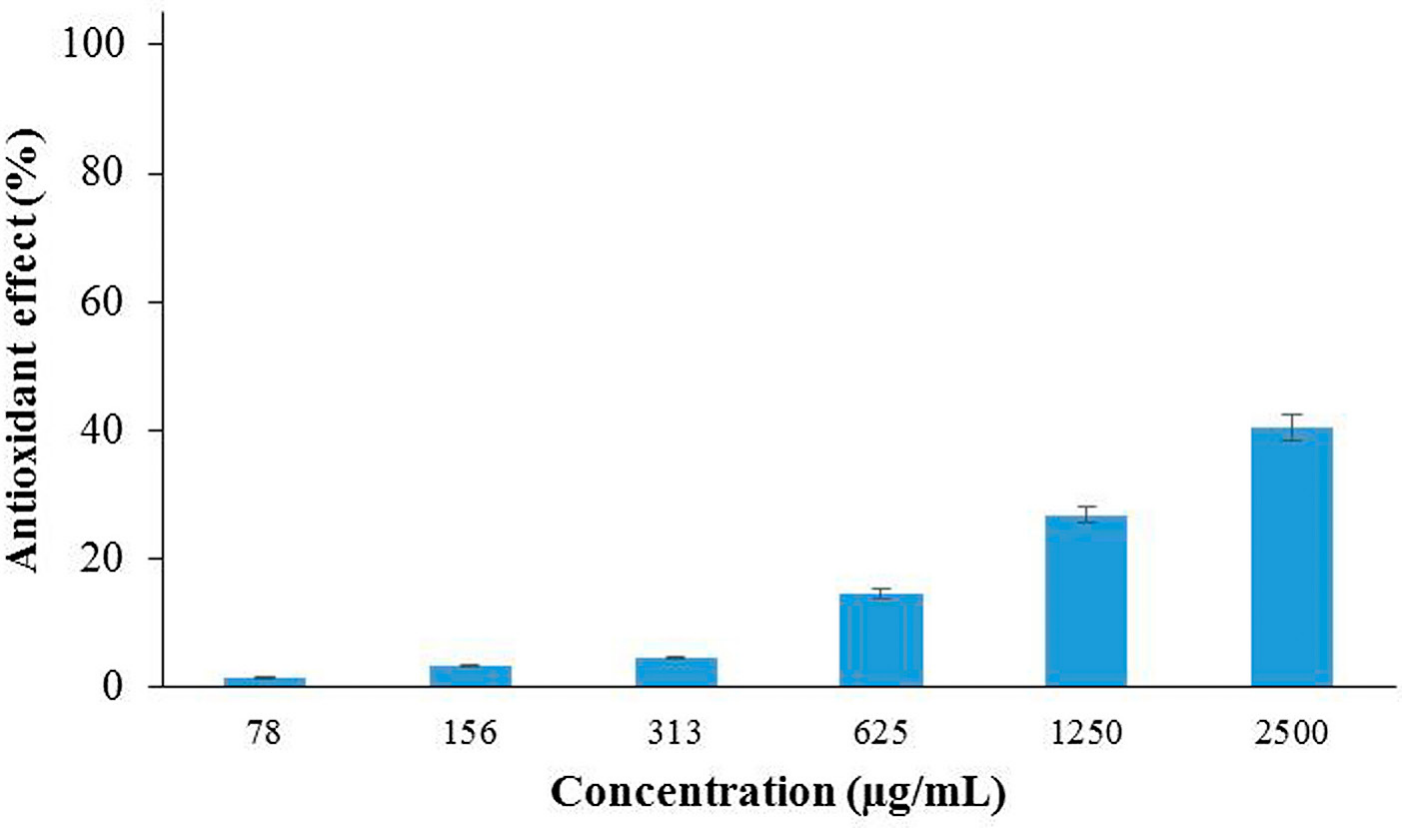
DPPH free‐radical scavenging activity in different concentrations of alginate nanoparticles containing *Zataria multiflora* and *Cuminum cyminum* EOs.

The antioxidant potency of the mixture of nanoparticles is shown in Figure 3. According to the obtained results, the concentration of 2500 μg/mL was the strongest DPPH free‐radical inhibition activity (40%). Recently, there has been growing attention to discovering antioxidant phytochemicals because they could hinder the expansion of free radicals and defend the human body against different illnesses (Mahmoud et al., 2021). In addition, phenolic compounds present in plants have been reported to contain potent antioxidant properties, which may assist in keeping cells from oxidative destruction triggered by active free radicals (Lin et al., 2009).

### 3.5 Chemical analysis of shrimp samples during cold storage

#### 3.5.1 Determination of pH changes

**FIGURE 4:**
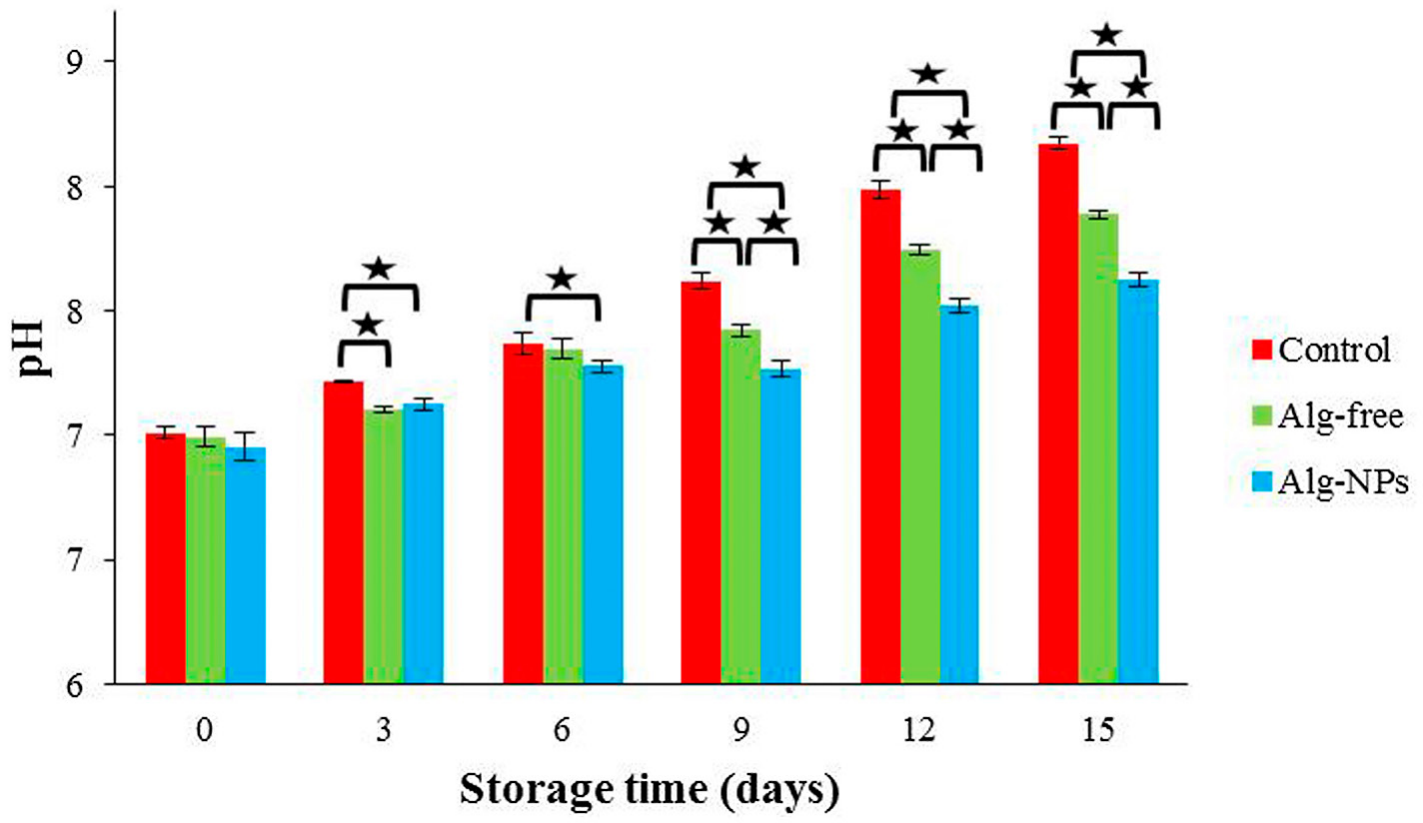
pH changes of shrimp samples during refrigerated storage. *p < .05.

Figure 4 shows the changes in pH value for shrimp samples during cold storage. The shrimp samples' initial pH (day 0) was 6.95–7.01. The pH values of all shrimp samples increased significantly during storage (*p* < .05). For instance, for the control group, pH increased significantly and reached 8.17 on day 15 of refrigerated storage, which was significantly higher than other samples (*p* < .05). However, this increase in the coated shrimp with alginate nanoparticles containing *Z. multiflora* and *C. cyminum* EOs (Alg‐NPs) was the lowest (from 6.95 to 7.62 on day 15); it is due to the antibacterial properties of alginate and EOs to control bacterial growth. The rise in shrimp pH is due to the activity of enzymes or bacteria and, subsequently, the aggregation of alkaline components (such as ammonia and trimethylamine) (López‐Caballero et al., 2007). Thus, it can be said that the application of alginate nanoparticles containing EOs diminished bacterial growth and subsequently decreased the endogenous alkalinizing reactions involved in limiting the shelf life of shrimp. The results of Yousefi, Azizi, et al. (2018), Yousefi, Farshidi, and Ehsani (2018), Rostami et al. (2017), and Jasour et al. (2015), which studied the effect of alginate, whey protein, and chitosan coating containing lactoperoxidase system on pH of chicken breast fillet, Pike‐Perch fillet, and fish, respectively, were in good agreement with our results. As the pH amounts of all shrimp samples (Control, Alg‐free, and Alg‐NPs) were lower than the acceptable level, even during the emergence of undesirable sensory characteristics in the sample, it could be said that the pH index is not a correct quality value (Farshidi et al., 2018).

#### 3.5.2 Assessment of TBARS changes

**FIGURE 5:**
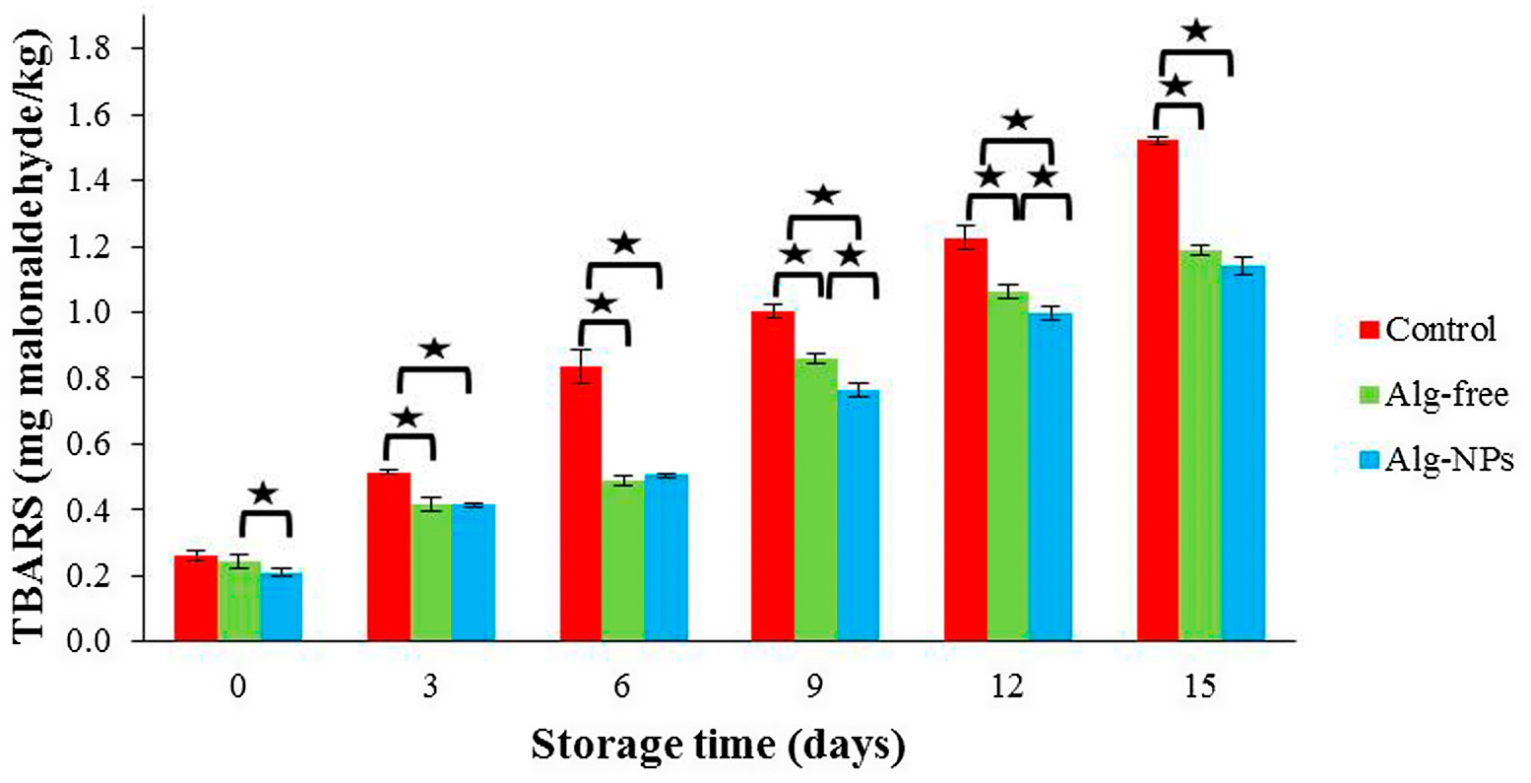
TBARS changes of shrimp samples during refrigerated storage. *p < .05.

The concentration of malondialdehyde (MDA), one of the secondary oxidation compounds generated from the decomposition of lipid hydroperoxides, determines the lipid oxidation rate. For the measurement of MDA, the thiobarbituric acid test is commonly used (Bensid et al., 2014). As shown in Figure 5, the TBARS value for all samples was between 0.21 and 0.26 mg MDA/kg shrimp at the early cold storage time. Similar to the pH value, the TBARS amounts of all shrimp samples increased progressively until the end of storage and reached 1.14, 1.19, and 1.52 mg MDA/kg in Alg‐NPs, Alg‐free, and control samples, respectively. During the oxidation process of unsaturated fatty acids, compounds such as hydroperoxides and peroxides are produced, resulting in MDA creation (Sun et al., 2017). It should bear in mind that lipid peroxidation is related to off‐flavor in products containing PUFA during storage (Karabagias et al., 2011). Lower TBARS value in Alg‐NPs treatment could be due to elevated concentrations of antioxidant components such as thymol and carvacrol in *Z. multiflora* EO and cuminic aldehyde in *C. cyminum* EO, which have a great antioxidant potential to inhibit lipid oxidation. They show their antioxidant activity by scavenging reactive oxygen species (ROS) and active chelating elements like iron by their functional moieties (Abbasi et al., 2021; Alipanah et al., 2022).

Huang et al. (2020) also reported that the TBARS content in control carbonado chicken samples was significantly higher than that in samples containing ε‐poly‐l‐lysine and rosemary extract during 15 days of cold storage (Huang et al., 2020). Criado et al. (2020) also reported that the application of alginate films containing cellulose nanocrystals (CNCs) was efficient in preventing lipid oxidation of chicken breast samples (Criado et al., 2020). Kilinc et al. (2009) reported that TBARS values less than 5 mg MDA/kg represent high‐quality food (Kilinc et al., 2009). All calculated TBARS contents in the present study were lower than this limit.

#### 3.5.3 Assessment of TVBN changes

**FIGURE 6:**
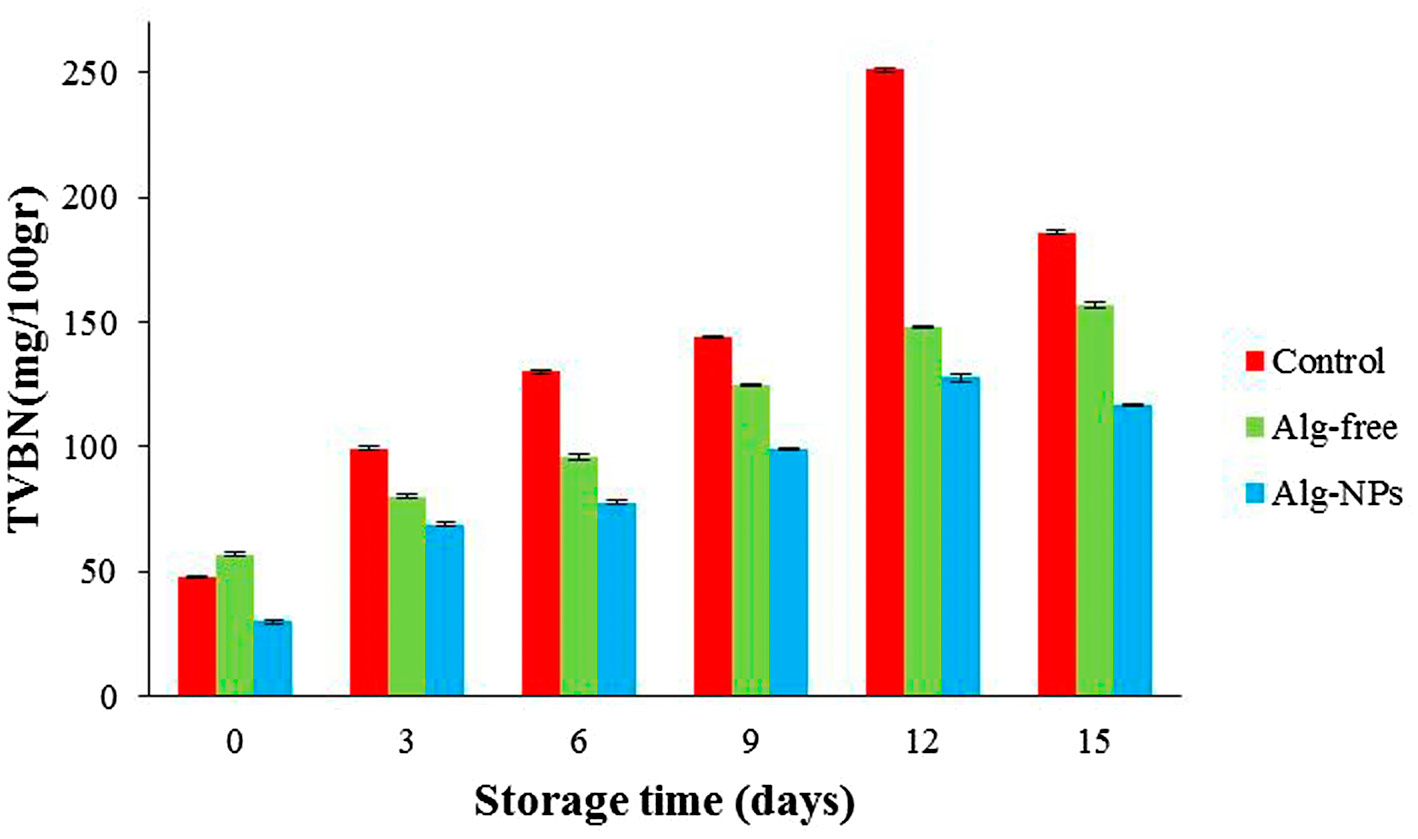
TVBN changes of shrimp samples during refrigerated storage (differences were significant at all times between all samples [*p* < .05], however, asterisks are not shown due to figure crowding).

Alterations of the TVBN are one of the indicators of shrimp deterioration due to the presence of high amounts of amino acids and soluble nitrogen in shellfish meat (Farshidi et al., 2018). Enzymes of microorganisms convert trimethylamine oxide (TMAO) to trimethylamine (TMA), which finally results in the generation of a volatile nitrogen base (Lu, 2009). The trend of TVBN amounts for shrimp samples is shown in Figure 6. As presented in this figure, the TVBN value of the shrimp samples was 29.75–48.04 mg/100 g in the early stage of storage. TVBN contents demonstrated an increasing trend in 15 days of refrigerated storage for all shrimp samples (the differences were significant at all times between all samples) (*p* < .05). It is worth noting that the TVBN amounts of our study were in good agreement with the changes in pH during cold storage (Figure 4). For instance, the highest amount of observed in the control sample in all time intervals during cold storage (48.04 and 185.9 mg/100 g on days 0 and 15, respectively). However, this value in Alg‐NPs samples was the lowest (at all sampling times) compared to control and Alg‐free samples, possibly due to the inhibition of the formation of TVBN, which increased from 29.75 to 117 mg/100 g during cold storage. Thus, it could be said that the antimicrobial agents (EOs) present in Alg‐NPs samples are greatly effective in the hindrance of the generation of the TVBN by microorganisms. Our result agreed with a study by Yousefi, Azizi, et al. (2018) and Yousefi, Farshidi, and Ehsani (2018), which observed that the alginate coating containing lactoperoxidase could inhibit the TVBN generation in chicken breast meat during storage (Yousefi, Azizi, et al., 2018; Yousefi, Farshidi, & Ehsani, 2018). Similar observations were also stated by Shokri et al. (2015). These observations showed that the assessments of TVBN can be a good indicator for evaluating the shrimp spoilage during storage at 4°C.

### 3.6 Microbiological analysis

**FIGURE 7:**
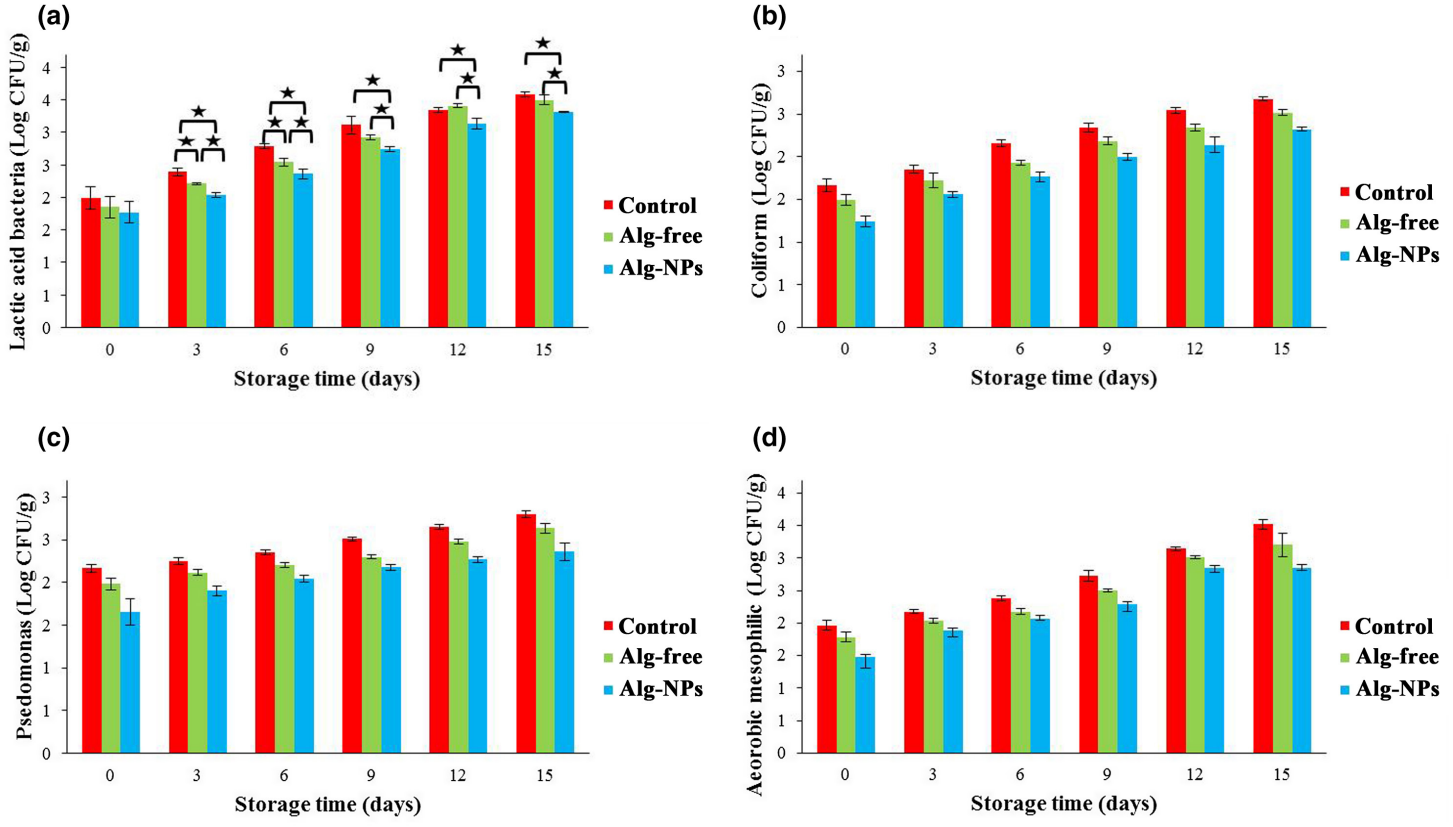
Changes in LAB (a), coliform (b), *Pseudomonas* (c), and aerobic mesophilic (d) counts of shrimp samples during refrigerated storage (in c and d, the differences were significant at all times between all samples [*p* < .05], however, asterisks are not shown due to figure crowding).

Food‐borne microorganisms are one of the most sources of deterioration in foodstuff (Hashemi et al., 2021). The antimicrobial properties of EOs on a variety of microorganisms have been revealed. Most of the antimicrobial potential of these compounds could be attributed to their phenolic compounds which involve in sensitization of the phospholipids present in the cell membrane, triggering a rise in permeability and leakage of crucial intracellular elements or damaging the bacterial enzyme structures (Chamanara et al., 2013). LAB, coliform, pseudomonas, and aerobic mesophilic bacteria were evaluated among all treatments during storage in a refrigerator at 4°C (Figure 7a–d). The trend of all bacteria counts was similar regarding all investigated shrimp samples since the antimicrobial effect of *Z. multiflora* and *C. cyminum* EOs is quite similar against the tested microorganisms. The mechanism of alginate nanoparticles of *Z. multiflora* and *C. cyminum* EOs against different kinds of bacteria are mostly related to that thymol, carvacrol, and cuminic aldehyde probably caused the breakdown of bacteria membrane, resulting in the cytoplasmatic seepage, cell lysis, and finally its damage (Hashemi et al., 2021; Li et al., 2020). Our results revealed that the alginate nanoparticles cannot decline bacterial growth efficiently while applied alone (Alg‐Free sample), but it limited bacterial contamination when the combination of *Z. multiflora* and *C. cyminum* EOs were added (Alg‐NPs sample). It has been revealed that nanoemulsified EOs has a higher antimicrobial effect in comparison with their typical emulsions due to the reduction in EOs particle size and higher diffusion of the active agents into the microorganisms (Dini et al., 2020) which confirm the present result. Similar trends were reported in previous studies, such as EOs nanoemulsion reducing microbial growth in food products (Dini et al., 2020; Li et al., 2020; Zhang et al., 2019, 2020).

Several reports also have shown synergistic and/or additive antimicrobial effects between different EOs (Abbasi et al., 2021; Lambert et al., 2001; Mojaddar Langroodi et al., 2021; Zarei et al., 2015). For example, Gibriel et al. (2013) reported that using a mixture of EOs (*C. cyminum*, *Z. multiflora*, and rosemary) showed a synergistic impact; so lower amounts of each EOs can be incorporated (Gibriel et al., 2013).

LAB grows slowly during cold storage and plays an important role in meat spoilage (Dehghani, Hosseini, Golmakani, et al., 2018; Dehghani, Hosseini, & Regenstein, 2018; Majdinasab et al., 2020). As depicted in Figure 7a, the LAB count of all samples increased with storage time, especially for the control sample (1.99–3.58 Log CFU/g), which showed the highest count in all time intervals. Initial LAB ranged from 1.77 to 1.99 Log CFU/g. After storage at the refrigerator (4°C) for 15 days, the total LAB reached 3.58, 3.50, and 3.32 Log CFU/g for control, Alg‐free, and Alg‐NPs samples, respectively. In comparison to the control sample, the coated samples (i.e., Alg‐free and especially Alg‐NPs) showed a lower number of LAB during cold storage (*p* < .05), which indicates the possible effect of edible coating to increase the shelf life of shrimp. For example, at the end of storage, the LAB samples coated with Alg‐Free and Alg‐NPs were 3.50 and 3.32 Log CFU/g. In all intervals, the lower number of LAB in Alg‐NPs samples indicates the high antimicrobial activity of *Z. multiflora* and *C. cyminum* EOs. To this end, the results showed that nanoparticle coating could effectively increase the shelf life of shrimp by reducing the total number of LAB. In the case of LAB number, the results of Mojaddar Langroodi et al. (2021), Mehdizadeh and Langroodi (2019), Rezaeifar et al. (2020), and Raeisi et al. (2015) studies (which investigated grape seed, lemon verbena, and propolis extract as well as *Z. multiflora* and oregano EOs) were similar to the present study. It is well known that Gram‐positive bacteria (like LAB) have more sensitivity to phenolic agents compared with Gram‐negative ones due to their differences in cell membrane characteristics (Majdinasab et al., 2020). The potent antimicrobial activity of EOs' components in the nanoscale is an effective factor in the inhibition of spoilage in the Alg‐NPs sample. The bacterial number in coated samples containing nanoparticles of EOs (Alg‐NPs) was less than in the control sample due to the antibacterial properties of natural EOs as well as the barrier effect of the coating agent (Ruelas‐Chacon et al., 2020). The nanoscale EOs could interact more efficiently with bacteria, due to the higher surface area, subsequently leading to cell death. Moreover, encapsulating EOs could enhance their stability, solubility, and dispersability and carry them effectively to specific sites with controlled release during cold storage (Zhang et al., 2019, 2020).

A similar trend was perceived for coliform growing during storage (Figure 7b). Initial coliforms ranged from 1.24 to 1.67 Log CFU/g. The uncoated shrimp (control sample) showed the highest increase in coliform counts during cold storage (1.67–2.68 Log CFU/g). Among the two coated samples, the Alg‐NPs samples had lower counts of coliform during cold storage (1.24–2.32 Log CFU/g), which displayed the best antimicrobial activity regarding inhibition of the coliform growth among other treatments (*p* < .05). In the case of coliform counts, the da Silveira et al. (2014) study results were similar to the present study. They reported that the *Laurus nobilis* EO could inhibit the growth of coliforms in fresh sausage stored at 7°C (da Silveira et al., 2014). Our results validate the outstanding antimicrobial activity of EOs‐loaded edible nanoparticles alginate coating for enhancing the microbial protection and shelf life of seafood products. The coliform‐defeating potential of EOs incorporated into edible coatings has also been informed by several researchers (Behbahani et al., 2020; Zhang et al., 2019, 2020). These agents have antimicrobial properties against a wide range of microorganisms. In fact, they could break down the outer membrane of Gram‐negative bacteria probably through mechanisms like the chelation of cations, leading to discharging of ATP energy supply. A decline in the pH slope within the cell membrane and a decrease in the intracellular level of potassium ions have been also perceived under the influence of phenolic compounds like carvacrol. These phenols act as an antimicrobial compound against both Gram‐negative and Gram‐positive bacteria probably via enhancing penetrability and fluidity of the cell membrane leading to the leakage of lipid and protein components (Dehghani, Hosseini, Golmakani, et al., 2018; Dehghani, Hosseini, & Regenstein, 2018).

Fresh shrimp is spoiled during cold storage mostly by Gram‐negative psychrotrophic bacteria (especially Pseudomonas species) (Dehghani, Hosseini, Golmakani, et al., 2018; Dehghani, Hosseini, & Regenstein, 2018). Pseudomonas species are one of the most important microflora of meat. They are recognized as particularly aerobic bacteria which could not survive in situations with inadequate oxygen (Behbahani et al., 2020; Chamanara et al., 2013). It is reported that the spoilage of meat products kept under aerobic‐refrigerated storage is mostly related to the growth and metabolic activity of Pseudomonas species, which are capable to decompose amino acids and glucose under cold storage (Behbahani et al., 2020; Chamanara et al., 2013). The proteolytic activity of this bacteria is probably responsible for meat spoilage and the following slime generation (Majdinasab et al., 2020). It is reported that when *Pseudomonas*'s number is between 7 and 8 Log CFU/g, they cause spoilage in raw meat (Mehdizadeh & Langroodi, 2019). The *Pseudomonas*'s growth in shrimp samples during cold storage is depicted in Figure 7c. The primary pseudomonas number varied between 1.65 Log CFU/g in the Alg‐NPs sample and 2.16 Log CFU/g in the control sample. Similar to previous bacteria, the count of Pseudomonas in all treatments increased during cold storage. However, the lowest and highest increase was observed in Alg‐NPs and control samples which determined 2.36 and 2.80 Log CFU/g, respectively, on day 15 of refrigerated storage. Therefore, alginate coating containing EOs showed high antimicrobial activity against the Pseudomonads. These results are also consistent with Farshidi et al. (2018) study investigating the influence of whey protein coating containing lactoperoxidase on shrimp quality and *Pseudomonas*'s growth inhibition (Farshidi et al., 2018). Moreover, in the study done by Mehdizadeh and Langroodi (2019), the number of Pseudomonas in chicken breast meat samples also increased during cold storage, and the samples coated with chitosan‐containing propolis extract and *Z. multiflora* EO displayed considerably lower counts, undeniably due to the antimicrobial effect of EOs during the storage period (Mehdizadeh & Langroodi, 2019). It is worth noting that the edible coating of alginate nanoparticles containing EOs was effective in limiting the oxygen distribution amount and prevented the development of this bacteria group which is the main psicrotrophic bacteria (Behbahani et al., 2020; Chamanara et al., 2013). These results are in good accordance with a previous study investigating the effect of the edible film based on chitosan‐containing nanoemulsion EOs on beef loins, lamb meat, and pork (Dini et al., 2020; Pabast et al., 2018; Zhang et al., 2019, 2020). The result verifies the intense antimicrobial activity of EOs due to the high concentration of thymol, carvacrol, and cuminic aldehyde (Majdinasab et al., 2020).

Figure 7d shows the count of aerobic mesophilic in all shrimp samples during 15 days stored in the refrigerator. Microbial contamination of fresh raw meat products including shrimp during cold storage is inevitable. As a result, the counting of mesophilic bacteria is mandatory in several safety regulations (Dehghani, Hosseini, Golmakani, et al., 2018; Dehghani, Hosseini, & Regenstein, 2018; Majdinasab et al., 2020). As presented in this figure, the number of this bacterial group also increased significantly (*p* < .05) with storage period in all treatments, especially in the control samples, which increased from 1.97 (on day 0) to 3.52 Log CFU/g (at the end of storage). Compared with the control sample, the coated samples showed a lower growth degree, which exhibits the probability of applying the active edible coating to prolong the shelf life of shrimp. The initial number of aerobic mesophilic was ranged between 1.48 and 1.97 Log CFU/g. After storage at 4°C for 15 days, the total number of this bacteria reached 2.84–3.15 Log CFU/g. Among the samples, the Alg‐NPs samples had a significantly lower number of aerobic mesophilic bacteria in all time intervals during cold storage (*p* < .05). Overall, these results presented that nanoparticle coating can effectively inhibit the total number of bacteria and increase the shelf life of shrimp. This pattern was in good agreement with the study's observations by Alparslan et al. (2016). They stated a lower increase in aerobic mesophilic in shrimp samples coated with gelatin containing oregano leaf EOs than control (Alparslan et al., 2016). Regarding the limit of 7 Log CFU/g, the acceptable level of aerobic mesophilic amount for fresh shrimp meat, the incorporation of both *Z. multiflora* and *C. cyminum* EOs into the alginate nanoparticle coating was efficient in inhibiting the microbial progress of shrimp through refrigerated storage (Huang et al., 2018). Regarding the results, using alginate nanoparticles rich in *Z. multiflora* and *C. cyminum* EOs raised the shelf life of shrimp due to hindering the bacterial growth in the sample, demonstrating the managed release of EOs of thyme and cumin from the nanoparticles, as their antimicrobial activity was proved in the in vitro trials (Behbahani et al., 2020; Chamanara et al., 2013). Similar findings related to the antimicrobial potential and shelf‐life extending capacity of EOs in nanoscale for different kinds of foods has been reported in several studies (de Oliveira et al., 2014; Dini et al., 2020; Homayonpour et al., 2021; Huang et al., 2020; Zhang et al., 2019, 2020).

### 3.7 Sensory evaluation

**FIGURE 8:**
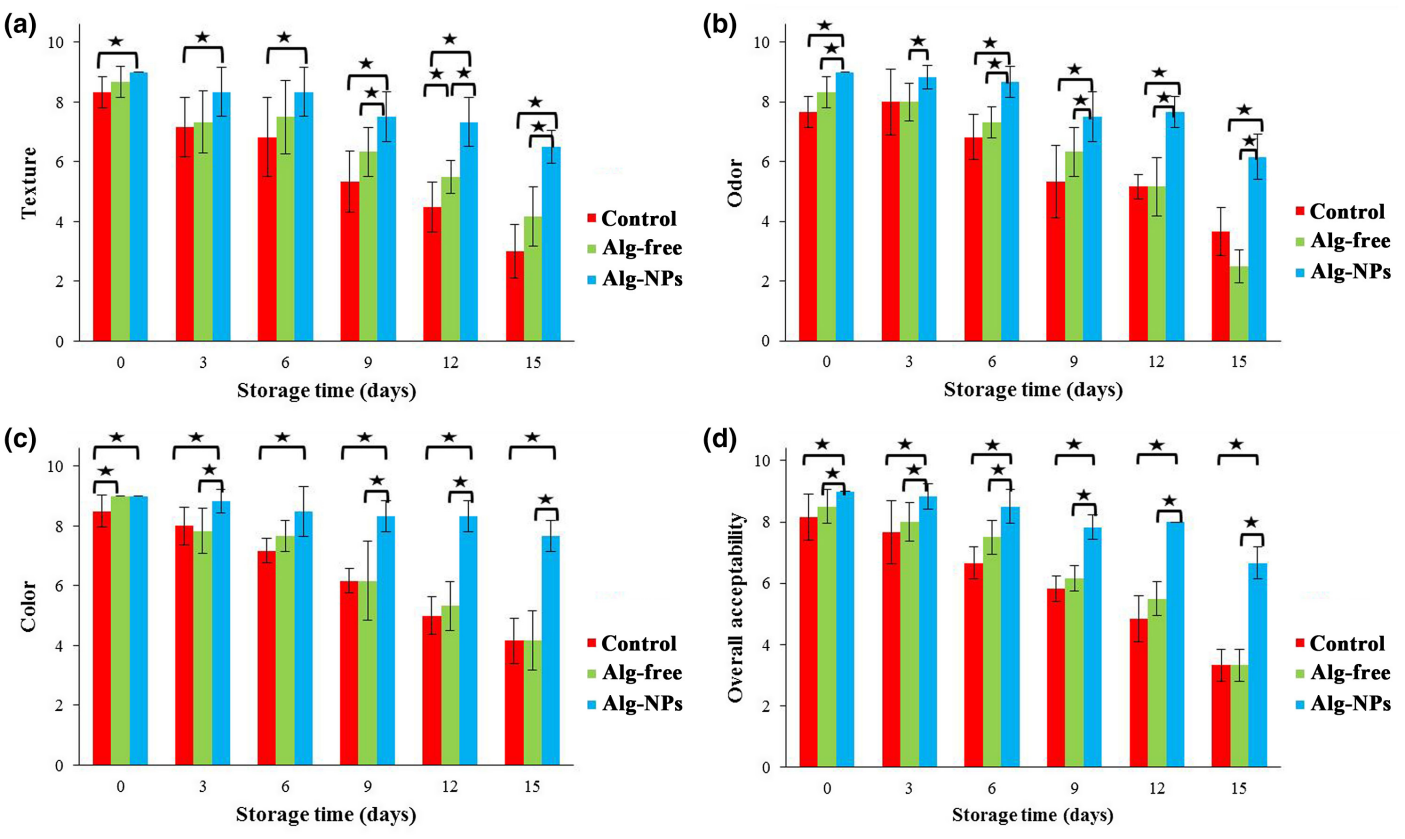
Changes in texture (a), odor (b), color (c), and overall acceptability (d) values of shrimp samples during refrigerated storage. *p < .05.

Figure 8a–d is illustrated the alterations in sensory characteristics (texture, odor, color, and overall acceptability) of all shrimp samples during refrigerated storage based on 9 points hedonic test. It is worth noting that scores lower than 7 are recognized as unacceptable for consumers (Mojaddar Langroodi et al., 2021). As illustrated in this figure, the organoleptic scores pointed out a significant reduction (*p* < .05) in all shrimp samples during 15 days of storage at 4°C, probably because of the microbial activities as well as chemical alterations. The sensory evaluation scores for all the shrimp samples were reduced during cold storage. The results showed that the lowest and highest reductions were observed in Alg‐NPs and control samples, respectively. Therefore, it could be noted that *Z. multiflora* and *C. cyminum* EOs addition, due to their antioxidant and antimicrobial properties, could have a protective effect against chemical and microbial alterations and subsequent adverse effects on sensory properties. In the case of Alg‐NPs, the results showed that the score of all parameters maintained higher than 7 until the 15th day, except for the color, which was lower than 7 after the 15th day of storage. However, the texture and odor of the control sample were taken to unacceptable scores after 9 days (5.33 and 5.33, respectively), while the color and overall acceptability were unacceptable after 12 days of storage (5 and 4.83, respectively). To this end, the shelf life of shrimp could be extended using Alg‐NPs containing *Z. multiflora* and *C. cyminum* EOs, by approximately 15 days. It is worth noting that the results of the sensory assessment are partly related to chemical and microbial evaluations. Because of the higher microbial number and lipid oxidation in the control shrimp samples, lower sensory scores were also observed in this sample compared with coated samples. Our results agreed with Zeng et al. (2005), Rezaeifar et al. (2020), and Liu et al. (2020) studies.

### 3.8 Melanosis assessment

**FIGURE 9:**
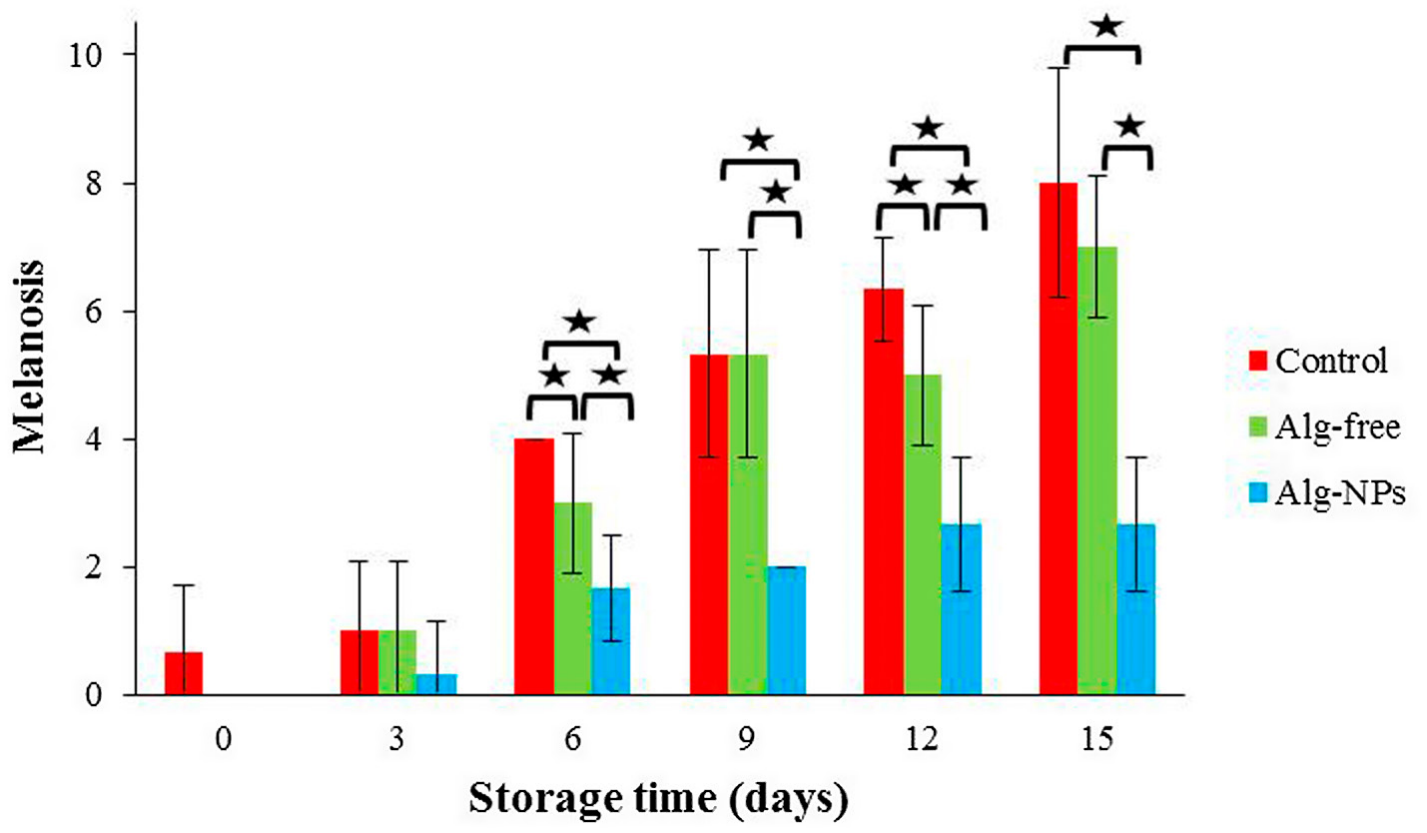
Melanosis changes of shrimp samples during refrigerated storage. *p < .05.

Figure 9 depicts the melanosis scores of shrimp samples; the melanosis in all shrimp samples was significantly increased during cold storage (*p* < .05). The melanosis in Alg‐NPs samples was significantly inhibited (*p* < .05), due to the effect of *Z. multiflora* and *C. cyminum* EOs, compared with the Alg‐free and control samples. The shrimp sample coated with Alg‐NPs was relatively unchanged during 15 days of cold storage (the scores changed from 0 to 2.67). On the contrary, black discoloration was formed in the control sample (the scores changed from 0.67 to 8). It is worth noting that *Z. multiflora* and *C. cyminum* EOs have antioxidant and antimicrobial properties (Gibriel et al., 2013; Osanloo et al., 2020).

Moreover, using EOs incorporated into coating materials may retard melanosis generation. Previous research have also proved that natural compounds hinder the generation of melanosis in shrimp (Perdones et al., 2014; Sun et al., 2014). It should be mentioned that the main enzyme involved in melanosis formation in shrimp is polyphenol oxidase. Our EOs, along with their antimicrobial and antioxidant traits, could probably block the activity of this enzyme and thus slow down the formation of melanosis (Sae‐Leaw & Benjakul, 2019).

## 4 CONCLUSION

This study determined that coating shrimp with alginate nanoparticles containing *Z. multiflora* and *C. cyminum* EOs was greatly efficient against developing several types of spoilage bacteria *vis*. LAB, coliform, pseudomonas, and aerobic mesophilic. This process also delayed lipid oxidation and sustained sensory characteristics, consequently increasing the fresh shrimp's shelf life during storage in the refrigerator (4°C). So alginate nanoparticles incorporated with *Z. multiflora* and *C. cyminum* EOs could be considered effective new active packaging for preserving fresh seafood under cold storage.

## CONFLICT OF INTEREST STATEMENT

The authors have no conflict of interest to disclose.

## Data Availability

The data that support the findings of this study are available on request from the corresponding author.
